# New Insights Into the Roles of Microglial Regulation in Brain Plasticity-Dependent Stroke Recovery

**DOI:** 10.3389/fncel.2021.727899

**Published:** 2021-08-05

**Authors:** Fang Yu, Tingting Huang, Yuanyuan Ran, Da Li, Lin Ye, Guiqin Tian, Jianing Xi, Zongjian Liu

**Affiliations:** ^1^Department of Neurology, University of Pittsburgh, Pittsburgh, PA, United States; ^2^Department of Anesthesiology, Westchester Medical Center, New York Medical College, Valhalla, NY, United States; ^3^Department of Anesthesiology, Renji Hospital, Shanghai Jiao Tong University School of Medicine, Shanghai, China; ^4^Department of Rehabilitation, Beijing Rehabilitation Hospital, Capital Medical University, Beijing, China; ^5^School of Materials Science and Engineering, Beijing Institute of Technology, Beijing, China

**Keywords:** stroke, microglia, neuroplasticity, rehabilitation, recovery, inflammation

## Abstract

Stroke remains the leading cause of long-term disability worldwide with significant long-term sequelae. However, there is no highly effective treatment to enhance post-stroke recovery despite extensive efforts in exploring rehabilitative therapies. Neurorehabilitation is recognized as the cornerstone of functional restoration therapy in stroke, where treatments are focused on neuroplastic regulation to reverse neural structural disruption and improve neurofunctional networks. Post-stroke neuroplasticity changes begin within hours of symptom onset and reaches a plateau by 3 to 4 weeks within the global brain in animal studies. It plays a determining role in spontaneous stroke recovery. Microglia are immediately activated following cerebral ischemia, which has been found both proximal to the primary ischemic injury and at the remote brain regions which have functional connections to the primary injury area. Microglia exhibit different activation profiles based on the microenvironment and adaptively switch their phenotypes in a spatiotemporal manner in response to brain injuries. Microglial activation coincides with neuroplasticity after stroke, which provides the fundamental base for the microglia-mediated inflammatory responses involved in the entire neural network rewiring and brain repair. Microglial activation exerts important effects on spontaneous recovery after stroke, including structural and functional reestablishment of neurovascular networks, neurogenesis, axonal remodeling, and blood vessel regeneration. In this review, we focus on the crosstalk between microglial activation and endogenous neuroplasticity, with a special focus on the plastic alterations in the whole brain network and their implications for structural and functional restoration after stroke. We then summarize recent advances in the impacts of microglial phenotype polarization on brain plasticity, trying to discuss the potential efficacy of microglia-based extrinsic restorative interventions in promoting post-stroke recovery.

## Introduction

**FIGURE 1:**
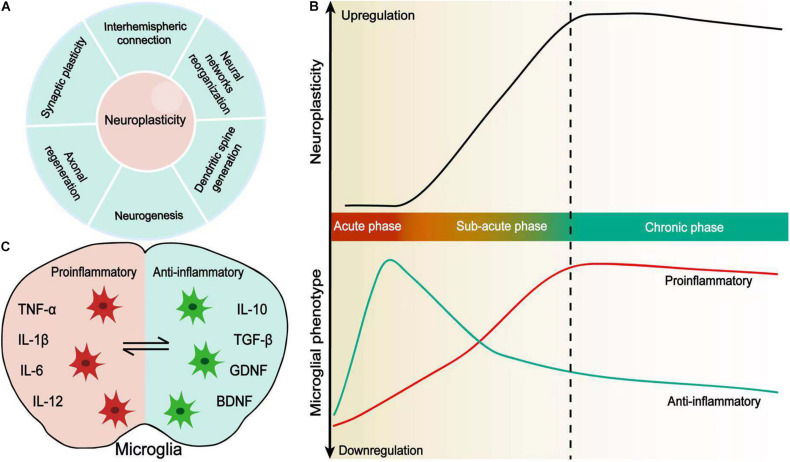
Microglial activation coincides with neuroplasticity after stroke. **(A)** Neuroplasticity is defined by synaptic plasticity, axonal regeneration, neurogenesis, dendritic spine generation, neural network reorganization, and interhemispheric connection. **(B)** Changes in spontaneous recovery and microglial polarization in a time-dependent manner. Microglial polarization evolves from the acute phase to chronic phase after stroke and coincides with enhanced neuroplasticity. **(C)** Pro-inflammatory and anti-inflammatory microglia. Resident microglia are activated and adopt a variety of different morphological and functional phenotypes, which secrete pro-inflammatory or anti-inflammatory cytokines following stroke.

Stroke is a devastating neurological disease, and it remains the leading cause of long-term disability worldwide, especially among the elderly (Dimyan and Cohen, 2011). Despite the therapeutic advance, especially in the acute phase, such as thrombolytic therapy and endovascular thrombectomy, a non-negligible proportion of stroke patients still suffer from long-term sensorimotor neurological deficits and/or cognitive impairment (Caleo, 2015). There is an urgent need for developing effective rehabilitation therapies after stroke, especially in the chronic phase. Endogenous neuroplastic changes and recovery following stroke are critical in helping patients to regain, partially for most cases, the neurofunction lost from primary ischemic brain injury. It involves functional and morphological reorganization of neuronal connections, excitability, axonal regeneration, neurogenesis, and dendritic spine rearrangement (Sandvig et al., 2018). Post-stroke neuroplasticity begins within hours of symptom onset and is maximally active at around 1 week after stroke (Xerri et al., 2014). It reaches a plateau by 3 to 4 weeks and could persist long term after stroke. Post-stroke neuroplasticity involves the global brain area, which might be associated with neuroinflammatory responses triggered by the primary ischemic injury (Shi et al., 2019). Novel therapies that enhance neuroplasticity after stroke are warranted to improve long-term neurofunctional recovery in ischemic stroke patients. Microglia, the resident immune cells in the central nervous system, are activated within minutes following ischemia and persist for days to weeks or even years following stroke (Ma et al., 2017). Microglial activation has been found in the ischemic core, peri-infarct zone, and remote brain regions that are functionally or anatomically connected to the primary ischemic injury (Gerhard et al., 2005). Activated microglia drastically alter their phenotypes and functions in a spatiotemporal manner upon ischemic insult. They are morphologically and functionally plastic with the change of microenvironments in different neurological pathologies (Lv et al., 2011), which coincides with post-stroke neuroplastic changes in a spatiotemporal manner (Figure 1). Post-stroke neuroplasticity changes provide the fundamental base for the microglia-mediated functional responses, which are involved in the entire brain network rewiring and brain repair (Price et al., 2006). Microglial activation participates in post-stroke plasticity, with effects on neurogenesis, axonal regeneration, reorganization of neural network and circuits, interhemispheric connections, etc. (Sandvig et al., 2018). The fact that microglia modulate global neuroinflammation after stroke and promote post-stroke neuroplasticity makes this special glial cell population an attractive candidate for targeting stroke recovery.

## Post-Stroke Microglial Activation and Global Brain Inflammation

Stroke affects almost all cellular elements within the brain and induce various signaling that occurs between or among different brain cell types. Microglia constitute up to 10% of the total brain cells and exert critical effects in maintaining brain homeostasis by phagocytosing cellular debris, presenting foreign antigens, and sensing pathological events such as ischemic stroke. Microglia also help to synchronize neuronal activity and plasticity, thus enhancing post-stroke neurofunctional recovery (Taylor and Sansing, 2013; Sandvig et al., 2018).

### Microglia Drive Global Inflammatory Response After Stroke

#### Remote Brain Areas Undergo Histopathologic Changes After Ischemic Stroke

Apart from the primary ischemic injury, histopathologic changes usually happen in non-ischemic remote brain regions that have synaptic connections with the primary ischemic lesion after stroke. For instance, in MCA territory ischemic stroke, neuronal death, reactive gliosis, and axonal degeneration have been found in the ipsilateral thalamus, substantia nigra (SN), and distal pyramidal tracts (PTs), which all took place outside of the MCA territory (Thiel et al., 2010; Zhang et al., 2012). Both anterograde PTs and retrograde thalamocortical fibers (Tamura et al., 1991) and nigrostriatal fibers (Forno, 1983) undergo neurodegenerative alterations after stroke. These pathological changes usually happen several days or weeks after stroke onset (Block et al., 2005) and have strong correlations with sensorimotor neurological deficits and post-stroke cognitive impairment (Domi et al., 2009; Yu et al., 2009). These histopathological changes in remote brain areas usually happen later than the initial ischemic infarction and start from the segments close to the lesion and extend to the remote segments (Arlicot et al., 2010). For example, Buss et al. (2004) found that axonal loss and myelin degradation first happen in the corticospinal tract of the cervical section 2 weeks after stroke onset and then progress to the lumbar segment at 5 weeks. While neurodegeneration descends in the spinal cord, activated microglia are present in the gray matter of the spinal cord contralateral to the primary brain lesion within 2 weeks after stroke (Schmitt et al., 2000; Zhang et al., 2012), and then from 5 weeks to 4 months, the number of activated microglia decreases in the gray matter but increases within the contralateral corticospinal tract (Schmitt et al., 2000).

#### Global Microglial Activation Following Stroke

Stroke elicits robust inflammatory reactions, and microglia are among the very first brain cells to react to the ischemic pathological changes, by responding to various damage-associated molecular patterns (DAMPs) released from the injured cells, such as adenosine, heat shock proteins, high mobility group box 1, and interleukin-33 (IL-33) (Shi et al., 2019). Together with astrocytes, endothelial cells, platelets, and other peripheral myeloid cells and lymphocytes, microglia elicit inflammatory responses in the acute phase predominantly at the infarct zones following ischemic stroke (Giraud et al., 2015; Neumann et al., 2015). However, studies on both human and animal experiments all identified that immune responses not only occur in remote areas from the initial ischemic zone in the acute phase but also persist into the chronic phase after stroke (Shi et al., 2019). In an endothelial-1-induced prefrontal infarct rat model, activated microglia were detected in the remote cortex and white matter at 28 days after ischemia, and this finding was associated with neuronal loss in these areas (Weishaupt et al., 2016). Consistently, activated microglia initially reside proximal to the ischemic brain lesion but gradually distribute to remote areas and finally reach a global distribution pattern within the brain. In animal study, remote microglial activity likely starts to occur around 2 days following stroke but maximizes at around 7 days after stroke (Morioka et al., 1993). Microglial infiltration within the whole brain and other remote areas, such as spinal cords, can persist for several months after stroke onset (Pappata et al., 2000; Gerhard et al., 2005; Price et al., 2006). In ischemic stroke patients, microglial activation can be observed as early as 3 days post-stroke and continues to persist for at least 150 days after human ischemic stroke, spreading from the connected region in both ipsilateral and contralateral hemispheres (Gerhard et al., 2005). In subcortical stroke patients, perilesional microglial activity increases soon after ischemic change but decreased over follow-up. However, remote microglia, especially within the PTs continue to be activated for at least 6 months (Pappata et al., 2000; Thiel et al., 2010). These anterograde tracts are highly likely to undergo Wallerian degeneration in the weeks or months following stroke (Thiel et al., 2010; Shi et al., 2019). Moreover, the extent of these anterograde microglial activity in the brainstem was positively associated with the extent of PT damage in both early (2 weeks) and chronic (6 months) periods after stroke (Thiel et al., 2010). PT integrity is highly correlated with residual motor function, while remote activated microglia in PT correlated with clinical outcome (Radlinska et al., 2010; Thiel et al., 2010). Increased microglial infiltration was detected in the thalamus ipsilateral to the stroke in patients in whom the infarcts affected thalamocortical or corticothalamic fibers. Moreover, brain areas with intense tracer binding were observed around subcortical lesions; along subcortical white matter tracts, including the internal capsule area and the corticospinal tract; and around the cortical areas (Ramsay et al., 1992; Gerhard et al., 2005; Price et al., 2006). These remote sites where microglia are activated have fiber tract connections to the original infarct area, and the microglial activation could be traced down to the spinal cord in patients with stroke involving the pyramidal tract (Schmitt et al., 1998). Such perturbations in the pan-brain area might be responsible for long-term neurofunctional deficit or cognitive impairment following stroke (Thiel and Vahdat, 2015).

The roles that microglia activation exert in remote brain areas to the initial ischemic injury are controversial, which indicates the dual and plastic effects that microglia/macrophage possess in the pathogenesis of secondary neural injury in remote brain areas. They may help to promote secondary fiber tract degeneration or be an inner responsive change to improve the fiber connection, according to the phenotype, activation timing, and brain location. The extent of PT damage determines the extent of microglial activation along the tract, and the microglial activity in the remote area is strongly associated with the favorable neurofunctional outcome, which implies that microglial activation around affect tracts might be beneficial in improving the repair of those axons/tracts undergoing Wallerian degeneration (Thiel and Heiss, 2011). Local microglia/macrophages produce osteopontin, protecting the secondary thalamic lesion through anti-inflammatory effects in the remote area to the ischemic lesion area (Schroeter et al., 2006). However, the remote responsive activity might be different from the initial injury, which needs to be taken into consideration when targeting microglia as a potential treatment option. For example, in the area of the infarct, phagocytic microglia mainly express the major histocompatibility complex antigen of type I (MHC I), while in the remote area, the non-phagocytic microglia express MHC II, representing distinct immune response profiles (Schmitt et al., 1998). The different response might be due to the phenotype switch upon dynamic interactions with surrounding brain cells, such as neurons, astrocytes, oligodendrocytes, or even endothelial cells, or simply depend on the microenvironments where they reside (Qin et al., 2019a). Moreover, the classical binary identification of microglia, as a pro-inflammatory phenotype and anti-inflammatory phenotype, is oversimplified. Microglia have overlapping functional states which need to be considered and can be switched from one to the other depending on the signal pathways that are activated. Some states of microglia are recognized as an intermediate phenotype, since they may produce both pro-inflammatory and anti-inflammatory markers (Chhor et al., 2013; Qin et al., 2019a). For example, M2a-like microglia increase the secretion of arginase, CD206, and chitinase 3-like 3, upon the inducement of IL-4 and IL-13, while M2b is considered as an intermediate state of microglia and produces both inflammatory and restorative markers. The M2c phenotype expresses D206, TGF-β, and CD163, which helps to enhance tissue remodeling and restoration after inflammation subsides (Chhor et al., 2013; Hu et al., 2015).

A holistic assessment and understanding of all involved mechanisms that microglia have in the damaged and recovering brain is of critical importance for outcome prediction and the identification of therapeutic targets. Because some of those processes are reversible, selectively targeting or strengthening the alternative pathway might be able to promote the recovery.

#### Possible Mechanisms by Which Microglia Respond in a Global Manner

Stroke is recognized as a global disease which elicits profound immune responses within the global brain and the body. The initial microglial response at perilesional sites promotes the inflammatory cascade within the brain. Systemic inflammatory response syndrome (SIRS) magnifies neuroinflammation and amplifies the inflammatory cascade, upon blood–brain barrier (BBB) disruption.

#### Peripheral Immune Cell Infiltration Into the CNS

Animal studies (Llovera et al., 2015; Jones et al., 2018) identified that peripheral immune cells infiltrate both the ipsilateral and contralateral hemisphere following stroke, which persists for at least 2 weeks and exaggerates the inflammation within the whole brain (Feng et al., 2017). Similar to the residing microglia which distribute in remote areas and the contralateral hemisphere, peripheral lymphocytic infiltration has been noticed in the thalamus area, promoting secondary neuronal damage distant from the initial ischemic injury (Jones et al., 2018). Ischemic stroke is a major contributor to vascular cognitive impairment or even dementia after years (Dichgans and Leys, 2017). Animal experiments found that lymphocyte B cells as well as immunoglobulin accumulate in the hippocampus area remote to the ischemic core and are associated with cognitive decline in stroke animals (Doyle et al., 2015; Doyle and Buckwalter, 2017). Peripheral immune cells, such as neutrophils, lymphocytes, and monocytes, migrate into the CNS and secrete a wide range of inflammatory mediators, which interacts with the microglia, potentially magnifying the inflammatory processes and secondary neurodegenerative changes at the whole-brain level. For example, in mouse ischemic stroke models, lymphocytes infiltrating into the whole stroke brain were complicated with inflammatory cytokines IL-1α, IL-1β, IFN-γ, TNF-α, and IL-6 at 2 weeks after stroke (Feng et al., 2017). The contralateral hemisphere was found to have significantly higher levels of IL-1β, IL-6, TNF-α, and TGF-β, compared with the brains of the sham group (Taylor and Sansing, 2013; Shi et al., 2019). When resting microglia meet with the infiltrating peripheral immune cells and/or inflammatory cytokines, they are more likely to be activated and present a pan-activation profile within the whole brain.

#### BBB Disruption Promotes the Global Brain Inflammation After Stroke

Just like a global infiltration of immune cells, the BBB disruption happens within the whole brain, thus promoting further inflammatory cascade, together with the immune cell and associated inflammatory markers. The BBB is critical in keeping brain homeostasis (Sweeney et al., 2019). Continuous endothelial cell deterioration is responsible for BBB disruption within the whole-brain areas, which may persist for several weeks after stroke onset (Merali et al., 2017). The initial mechanism of BBB breakdown is thought to be associated with the matrix metalloproteinases (MMPs), which degrade the endothelial cell, as well as the junctional connections among the endothelial cells (Nian et al., 2020). Vascular cell adhesion molecule (VCAM-1) is another inflammatory marker that promotes the adhesion and migration of peripheral immune cells, magnifying the inflammation and disrupting BBB integrity (Shi et al., 2019). Both animal and human studies confirmed a global vascular response and BBB breakdown after ischemic stroke, which may happen as early as 24 h after stroke onset (Quenault et al., 2017; Villringer et al., 2017). In the chronic phase of ischemic stroke, elevated levels of MMPs are also present in the non-intact hemisphere and continues to induce BBB breakdown and global inflammation (Zinnhardt et al., 2015).

#### The SIRS and Brain–Body Axis

Systemic inflammatory response syndrome has been found in both ischemic stroke and hemorrhagic stroke, and it can persist for at least 90 days after stroke onset in clinical studies (Saand et al., 2019; Tsai et al., 2019). A clinical study included 24 stroke patients and evaluated their peripheral immune profiles, which showed different immune responses after stroke. Key elements of three immune response phases were also identified: an acute phase as early as post-stroke day 2 with increased signal transducer and activator of transcription 3 (STAT3) in innate immune cells, an intermediate phase starting from day 5 with cAMP response element-binding protein (CREB), and a late phase at around 3 months after stroke with neutrophils and IgM-positive B cells (Tsai et al., 2019). A continuous activation of systemic immune cells and chronic global vascular activation as well as BBB disruption interactionally affect each other, possibly contributing to global inflammation, impairing long-term neurofunctional recovery, and promoting cognitive decline in stroke patients (Mijajlovic et al., 2017).

## Microglial Activation With Brain Plasticity After Stroke

It needs to be recognized that a stroke not only causes neuronal loss in the infarct area but also disrupts the entire brain networks, resulting in a widespread dysfunction and impairments, including sensorimotor dysfunction, cognitive impairment, and mood disorders. Microglia might have profound implications in post-stroke neuroplastic changes and represent a potential candidate for therapeutic targets.

### Microglial Activation and Post-stroke Neuroplasticity

Neuroplasticity refers to the brain’s intrinsic ability to reorganize its function and structure after brain injury. It involves both spontaneous rewiring of neural networks and circuits, as well as functional responses in neurogenic niches. The partial rewiring of survival neural networks and recruitment of intact synapses usually happen at the contralateral, ipsilateral to the brain ischemic lesion, as well as other remote areas (Alia et al., 2017). For example, animal studies showed that ischemic brain injury which affects the primary motor or somatosensory cortex causes early reorganization of regional representation of spared neural circuits within the perilesional brain area and a redistribution of neuronal receptive fields (Brown et al., 2010; Xerri et al., 2014). Post-stroke neuroplastic alteration is crucial in compensating or substituting for the primary lesion-induced functional impairment (Sandvig et al., 2018). Clinical studies identified that these histological and functional changes within the CNS account for spontaneous recovery in some stroke patient populations at 1 to 3 months after stroke onset, with improvement in both cognitive function and voluntary movement (Grefkes and Ward, 2014). The mechanisms underlying this neuroplastic process in post-stroke recovery involve various aspects of changes, such as the release of anti-inflammatory cytokines, neurogenesis, angiogenesis, axonal rearrangement, synaptic remodeling, the extracellular matrix (ECM), and microenvironment adaptation (Sandvig et al., 2018), as well as the activation of endogenous neurogenesis (Frisen, 2016). Another major component of post-stroke neuroplasticity change is the neurotransmission disorder. The unbalance in excitatory and inhibitory neurotransmission production within the ischemic core and peri-ischemic lesion affects the remapping of the neural network in both the acute phase and chronic phase after stroke (Schmidt et al., 2012). Microglia might play a critical role in the post-stroke neuroplastic process mentioned above and may represent a potential therapeutic target to enhance long-term stroke recovery.

In intact CNS, microglia scan the microenvironment within the whole brain and respond to pathological injuries. The distribution of microglia varies according to different brain areas in adult mouse: a larger proportion of microglia resides in the gray matter compared to white matter; meanwhile, more abundant microglia are found in the substantia nigra, basal ganglia, hippocampus, and olfactory telencephalon, when compared with the cerebellum and brainstem (Lawson et al., 1990). However, human brain microglia exhibit an opposite distribution profile, with more microglia residing in the white matter and lower proportion in the gray matter (Thiel and Heiss, 2011). Overall, the distinct differences in the distribution and dual phenotypic characteristic of resident microglia in the brain indicate high sensitivity to microenvironmental changes and different activities and functions upon brain injury at different brain areas. Microglia are critical in shaping and maintaining the functional neural network and participate in synapse formation. In physiological states, microglia are physically engaged in both presynaptic and postsynaptic areas, help to regulate the protrusion of elimination of dendritic spines, and process the signaling information. On the other hand, microglia are of great importance in regulating neurogenesis within the endogenous neurogenic niches such as the subventricular zone (SVZ), where neural progenitor cells (NPCs) differentiate (Sandvig et al., 2018). Upon ischemia, microglia change in activity and morphology in response to the injury and contribute to reorganizing the neural network and synapse. Studies showed that microglia respond to inflammation and mediate inflammation in such processes, while modifying neural activity and regulating the synaptic function.

### Microglia With Neurogenesis

Neurogenesis is critical in neural network restoration and remodeling after stroke (Xerri et al., 2014). Neuroblast production is increased in the stroke brain, where microglia are located, and they might play both detrimental and beneficial roles in neurogenesis (Ekdahl, 2012; Shigemoto-Mogami et al., 2014; Dos Santos et al., 2021). By releasing IL-4, microglia enhance NPC activity and promote neuroblasts to differentiate into neurons (Walton et al., 2006). The same effect has been noticed in aged mouse brain, where microglia interact with NPCs and promote their activities (Ma et al., 2017). However, microglia can also inhibit NPC proliferation through NO expression (Carreira et al., 2014). Animal studies also identified that microglial inhibition increases the number of newborn neural cells and enhances neurogenesis in the subgranular zone, which helps to rebuild the neural network (Sierra et al., 2010). Pretreatment and post-treatment with microglial inhibition medications, such as minocycline and indomethacin, enhanced the neurogenesis in the SVZs in stroke animals (Hoehn et al., 2005; Liu et al., 2007), while there are also other studies that proved that the number and activity increase of microglia in the peri-infarct zone also enhanced post-stroke neurogenesis (Ma et al., 2017). It is believed that microglia undergo dual activity and morphological changes in a spatiotemporal manner, depending on different neuropathologies and resultant micro-milieus, which may account for these controversial findings.

Microglial activation by different pathways exhibits different effects on neurogenesis. NPCs also participate in regulating microglial activity; they guide microglial proliferation and migration into the neuroblast niche (Ma et al., 2017). Microglia undergo a phenotype switch depending on the pathological stimulus or post-injury time points and exert different effects on neurogenesis (Hu et al., 2015). Destructive or unfavorable pro-inflammatory microglia produce proinflammatory factors, such as TNF-α, IL-6, IL-1β, ROS, and MMPs, aggravating neuronal death and impairing neurofunctional recovery in long-run periods, while anti-inflammatory microglia secrete TGF-β, IL-4, IGF-1, IL-10, and BNDF; promote basal neurogenesis; and enhance recovery (Hu et al., 2015). This so-called “angel or demon” characteristic of microglia could partly account for the dual function we observed in different studies.

Microglia play a dual role in neurogenesis, so investigations on when and how microglia influence neurogenesis will be helpful for developing the understanding of brain injury and repair after ischemic stroke (Ma et al., 2017).

### Microglia With Axonal Regeneration

Post-stroke neurogenesis alone cannot ensure functional recovery because axonal regrowth and the regeneration of the neural network are required (Kitayama et al., 2011). Neurons have very limited potential to initiate axonal regeneration after injury. The intrinsic capacity of neurons to grow is suppressed in order to stabilize the synaptic transmission and the interneuronal communications (Omura et al., 2015). However, microglia and macrophages promote axonal sprouting in the wound margin following brain injury (Batchelor et al., 2002). They produce local trophic gradients BDNF and GDNF, which stimulate axonal sprouting toward the wound edge. This axonal sprouting-promoting effect might help to limit the injury, but whether peri-wound axonal sprouting plays critical roles in neural network rebuilding is not sure yet.

Microglial phenotypes play different roles in regulating axonal regeneration (Hu et al., 2015). The pro-inflammatory microglia/macrophages inhibit axonal regeneration (Kitayama et al., 2011), while the anti-inflammatory phenotype is crucial for axonal regeneration. Anti-inflammatory cytokines such as IL-10 and oncomodulin are found to be essential in promoting axonal regrowth and recovery after SCI and optic nerve damage. Most of the studies were performed in animals; however, the human brain has a higher density of microglia in the white matter compared to the gray matter, and it is believed that they engender crucial roles in axonal modifications after ischemic stroke. Further investigations are warranted (Hu et al., 2015).

### Microglia With Synaptic Plasticity

Synapses are the key elements of neural circuits underlying normal brain function (Gould et al., 2019). Stroke disrupts neuronal networks including structural impairment and functional dysfunction in synapse. Surviving neurons in the peri-infarct regions are found to undergo spontaneous synaptic remodeling after stroke (DeBoer et al., 2021). Synaptic plasticity is correlated with outcomes of ischemic stroke (Qin et al., 2019b; Yang et al., 2020a). Rehabilitative therapies such as exercise training (Nie and Yang, 2017), enriched environment (Lin et al., 2021b), and transcranial magnetic stimulation (TMS) (Yang et al., 2020b) may restore the sensorimotor function via the enhancement of synaptic plasticity following stroke. Together, synaptic plasticity plays a crucial role in functional recovery after stroke. Microglia are shown to participate in synaptic plasticity through its crosstalk with neurons (Madinier et al., 2009; Yang et al., 2020a). Post-stroke microglial activation exerts a beneficial effect on synaptic plasticity-related proteins (synaptophysin and GAP-43) *via* the release of BDNF (Madinier et al., 2009). Knockout of BDNF in microglia alleviates learning-dependent synaptic formation (Parkhurst et al., 2013). The BDNF also upregulates the insertion of α-amino-3-hydroxy-5-methyl-4-isoxazole propionic acid)-type glutamate receptors (AMPA), thereby enhancing synaptic efficacy (Turrigiano, 2008). These studies indicated crucial roles of BDNF in microglia-mediated synaptic plasticity. Anti-inflammatory microglia are known to produce neurotrophic factors like BDNF, glial cell line-derived neurotrophic factor (GDNF), IL-10, and TGF-β. TWS119, a Wnt/β-catenin pathway activator, can modulate microglial polarization to an anti-inflammatory phenotype following ischemic stroke and strengthen neural plasticity and angiogenesis in the peri-infarct cortex, thus facilitating neurological recovery (Song et al., 2019). The CD200/CD200R pathway contributes to spontaneous functional recovery by enhancing synaptic plasticity via suppression of pro-inflammatory microglial polarization (Sun et al., 2020). Pharmacologic inhibition of TREM-1 with the synthetic peptide LP17 can potentiate synaptic plasticity in the hippocampus, resulting in reduced long-term functional deficits. The underlying mechanism might be associated with the inhibition of microglial pyroptosis/activation-induced neuroinflammation (Xu et al., 2019). Although there is increasing experimental evidence showing that microglial polarization-induced neuroinflammation is involved in post-stroke synaptic plasticity, the exact mechanisms are not fully understood. It may trigger more effective approaches for treating ischemic stroke patients to improve their neurological behavior if the molecular mechanisms can be elucidated.

### Microglia With Dendritic Spine Generation

In the condition of ischemic stroke, the infarct core fails to regain its fine dendritic structure after reperfusion (Li and Murphy, 2008; Murphy and Corbett, 2009). The dendritic spines of the surviving neurons in the ischemic penumbra undergo degeneration because of reduced blood supply; however, the loss of dendritic structures will partly be reversed when reperfusion occurs. This is where dendritic spine generation will occur to regain connectivity with axonal sprouting during the subacute and chronic phases of stroke. Stem cell factor (SCF) and granulocyte–colony-stimulating factor (SCF + G-CSF) at the chronic phase of experimental stroke can enhance dendritic spine formation and axonal sprouting in the cortex adjacent to the infarct cavity, indicating neural circuit rewriting (Cui et al., 2016). Previous studies reported that the extent of dendritic spine loss was associated with acute activation of microglia after transient cerebral ischemia (Ju et al., 2018; Hou et al., 2020). Metformin treatment can inhibit microglial hyperactivation and increase dendritic spine density via activating cerebral AMPK in rats after cardiac I/R injury (Leech et al., 2020). CD200 fusion protein (CD200Fc) treatment restrained microglial activation and release of pro-inflammatory cytokines and further recovers dendritic spines after stroke (Sun et al., 2020). Collectively, increases in dendritic spine production combined with axonal sprouting after stroke can be thought of as remodeling processes that help to compensate for lost structural circuits. The molecular determinants of dendritic remodeling are only partly known. Further investigations on potential mechanisms of dendritic spine generation with the inhibition of microglial activation after stroke are warranted.

### Microglia With Interhemispheric Connections

In stroke patients, inhibitory projections from the damaged hemisphere to the contralesional hemisphere are reduced, resulting in a disinhibition of the homotypic area in the contralesional hemisphere (Rehme and Grefkes, 2013). Therefore, interhemispheric connection is greatly weakened after stroke. It is thought that enhanced cortical network connectivity is generally associated with improved stroke outcomes. Sadler et al. (2020) found that short-chain fatty acid (SCFA) treatment at the chronic phase after stroke induced a reduced transhemispheric inhibition using the optogenetic imaging approach, indicating an increase in interhemispheric connectivity. The transcriptomic analysis showed that most genes are associated with microglial activation to be regulated by SCFAs. Thus, it is hypothesized that the SCFAs’ effect on interhemispheric connections might be indirectly mediated via microglial activation. This study provided insights into the crosstalk between interhemispheric connections and microglial function; however, the exact interactions should be further clarified.

### Microglia With Remyelination

The white matter is composed of myelin sheaths and enwrapped axons (Reiche et al., 2019). The myelin is wrapped around the internodes of axons, thereby facilitating saltatory conduction of neurological signals. The white matter is more susceptible to ischemic insult and more severely damaged compared with the gray matter, due to limited blood flow and restricted collateral circulation (Wang et al., 2016b). It has been reported that stroke outcomes were negatively correlated with white matter lesion (Zheng et al., 2021). Microglia-mediated neuroinflammation has a dual impact on the spontaneous repair of the white matter following stroke (Ma et al., 2017). Pro-inflammatory microglia aggravate stroke-induced oligodendrocytes/OPC death and suppress remyelination through pro-inflammatory cytokine secretion (Sierra et al., 2010). By contrast, regulating microglial polarization toward an anti-inflammatory phenotype can promote the repair of impaired white matter via release of beneficial factors including TGF-β and IL-10 (Ma et al., 2017; Qin et al., 2019a; Shao et al., 2021). More recent evidence shows that intracerebral infusion of regenerative microglia-derived extracellular vesicles (EVs) restores protective microglial/macrophage functions, limiting their senescence during the post-stroke phase, and enhances the maturation of OPCs at lesion borders, resulting in ameliorated neurological functionality (Raffaele et al., 2021). Cornel iridoid glycoside (CIG) treatment was reported to decrease microglial activation in the corpus callosum following stroke, accompanied with reduced white matter lesions and demyelination, increased myelin basic protein expression, and increased number of mature oligodendrocytes (Wang et al., 2019).

### Microglia With Angiogenesis

As a self-repair mechanism, angiogenesis plays an important role in long-term functional improvement following stroke (Li et al., 2014). Post-stroke angiogenesis can provide the vascular niche, which is required for the survival and migration of newborn neurons (Palmer et al., 2000). Of note, microglia exert crucial impacts on angiogenesis following stroke (Zhu et al., 2021). VEGF secreted from anti-inflammatory microglia is known to promote angiogenesis following ischemic stroke (Xie et al., 2013). Astragaloside IV (AS-IV) can enhance angiogenesis and functional recovery, at least partially through switching microglia/macrophages toward the anti-inflammatory phenotype in a peroxisome proliferator-activated receptor (PPAR)-γ-dependent manner after cerebral ischemia (Li et al., 2021). Berberine also was shown to promote angiogenesis through modulating anti-inflammatory microglial polarization *via* the AMPK pathway (Zhu et al., 2019). Besides, Noggin can regulate the macrophage/microglial polarization from the anti-angiogenic pro-inflammatory phenotype to pro-angiogenic anti-inflammatory phenotype after stroke (Shin et al., 2014).

### Microglia With BBB Disruption and Preservation

Blood–brain barrier disruption is a major contributor in the pathogenesis of ischemic stroke. It occurs as early as 12–24 h after ischemia and can persist for days (da Fonseca et al., 2014). A large body of literature justified that microglial activation contributes to BBB disruption in significant ways. Microglia accumulate and rapidly undergo morphological and functional changes, with cellular protrusions toward blood vessels, primarily enhancing BBB disruption. However, microglial inhibition either by CX3CR1 knockout or minocycline successfully reversed the effect, which confirms the role of microglia in post-stroke BBB dis-integrity (Machado et al., 2009; Jolivel et al., 2015; Ma et al., 2017). Multiple levels of mechanisms were endorsed to underlie microglial activation-induced BBB disruption, such as oxidative stress; cytotoxic mediator release (HIF-1, NF-κB, metalloproteinase, and pro-inflammatory cytokines IL-1β, IL-6, and TNF-α); and interactions with other BBB components, such as hindering the function of astrocyte and decreasing BBB integrity.

However, microglia possibly play a protective role in attenuating BBB disruption via secretion of progranulin, which is involved in reducing BBB disruption and brain edema after ischemic stroke (Egashira et al., 2013; Jackman et al., 2013; Ma et al., 2017). Therefore, the causal relationship between microglial activation and BBB disruption is still elusive. The effect of microglial activation on BBB disruption remains to be further explored.

## Microglia-Based Treatment Targeting Post-Stroke Neuroplasticity

**FIGURE 2:**
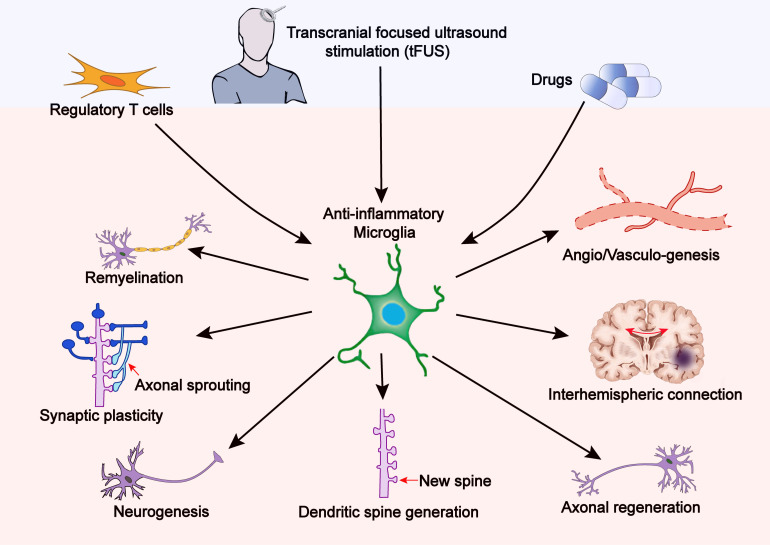
Therapy-induced anti-inflammatory microglia promote brain plasticity after stroke. Treatments with tFUS, Tregs, or certain drugs can shift microglial polarization toward the anti-inflammatory phenotype, thereby reinforcing brain plasticity including synaptic plasticity, axonal regeneration, neurogenesis, dendritic spine generation, neural network reorganization, and interhemispheric connection.

As stated earlier, microglia are crucially involved in brain plasticity and spontaneous functional recovery after stroke (Figure 2). However, the endogenous recovery after stroke is usually insufficient to significantly improve long-term neurofunctional outcomes. Therefore, therapeutic strategies that enhance post-stroke brain repair and functional recovery are of significant clinical importance. In this section, we aim to discuss microglia-based therapies that target neuroplasticity following stroke.

### Pharmacological Therapies

During the past several decades, numerous studies have demonstrated that pharmacological therapies can enhance brain plasticity and subsequently promote neurofunctional recovery via regulation of microglia-mediated neuroinflammation. Anti-inflammatory drugs targeting microglial polarization combined with rehabilitative therapy are more useful for tissue repair and functional recovery (Alam et al., 2016). In most cases, microglia-based pharmacological treatment has the most potential to produce a helpful microenvironment to reinforce neurorehabilitation after stroke. For instance, repetitive transcranial magnetic stimulation (rTMS) combined with minocycline treatment was shown to augment functional recovery by enhancing neuroplasticity and inhibiting microglia-mediated pro-inflammatory responses after stroke (Alam et al., 2016). Hydrogen-rich saline treatment restores the expression levels of synaptophysin and postsynaptic density protein 95 (PSD-95) via inducing anti-inflammatory microglial polarization, primarily through AMPK activation and NF-κB pathway inhibition following hypoxia–ischemia injury (Chu et al., 2019). GJ-4 (a natural extract from *Gardenia jasminoides* J. Ellis) improves memory impairment of stroke-induced vascular dementia rats by upregulating synaptophysin and PSD-95 expressions. The underlying mechanism is that GJ-4 suppresses microglia-mediated neuroinflammatory responses via inhibition of the Janus kinase 2 (JAK2)/signal transducer and activator of transcription 1 (STAT1) pathway (Liu et al., 2021a). Early inhibition of the Toll-like receptor (TLR)-4 pathway with TAK-242 (a TLR4-specific antagonist) administration improves the outcomes of neonatal hypoxic–ischemic brain damage though suppressing microglial activation and enhancing synaptic plasticity (Tang et al., 2019). 5Z-7-Oxozeaenol (a selective TAK1 inhibitor) significantly reduces axonal injury at the acute stage and hinders neuroinflammation at the subacute stage by shifting the microglial phenotype toward an inflammation-resolving state following stroke (Liu et al., 2021b). Treatment with a combination of Niaspan and simvastatin significantly improves functional outcome and increases axonal density, which is associated with reduced microglial activation after stroke (Shehadah et al., 2010). Ki20227 (a tyrosine kinase inhibitor) treatment reduces microglial activation, accompanied by a significant reduction in dendritic spine loss and behavioral deficits after transient global cerebral ischemia (Hou et al., 2020). Administration of curcumin (Liu et al., 2017) or melatonin (Liu et al., 2019c) indirectly decreases ischemic brain damage *via* inhibition of pro-inflammatory microglial polarization. Taken together, certain pharmacotherapies repair ischemic brain damage and improve long-term outcomes, which is associated with the enhancement in microglial regulation-mediated neuroplasticity.

### Regulatory T-Cell Therapy

The peripheral T cells infiltrate into the brain after ischemic stroke and interact with microglia (Wang et al., 2016a). CD4^+^ T cells, a subtype of T lymphocytes, exert important effects on brain damage and functional outcomes following stroke. They are differentiated into four subpopulations including T-helper 1 (Th1), Th2, Th17, and regulatory T cells (Tregs) with different functions (Theodorou et al., 2008). Among them, Th1 cells promote ischemic brain damage through transforming microglial polarization toward the pro-inflammatory phenotype (Tschoe et al., 2020). Th17 cells release IL-17, IL-21, and IL-22, which further promote pro-inflammatory microglial polarization and enlarge brain damage (Liu et al., 2019b). By contrast, Th2 cells play a protective role in the development of stroke. They secrete anti-inflammatory cytokines and further trigger anti-inflammatory microglial polarization after stroke (Korhonen et al., 2015). Thus, pro-inflammatory microglia-mediated secondary injury is inhibited, while brain repair and functional recovery are enhanced. Tregs are a minor subpopulation of CD4^+^ T cells, defined by the expression of signature proteins including CD25 and Foxp3 (Ferreira et al., 2019). Tregs begin to infiltrate into the infarct areas at 7 days and continued to be high in number up to at least 1 month after stroke insult (Ito et al., 2019). The accumulating Tregs are considered to promote post-stroke functional recovery by astrogliosis inhibition and/or neurogenesis induction (Wang et al., 2015). Hu et al. reported that the adoptive transfer of synergic Tregs confers BBB preservation and promotes long-term outcomes following ischemic stroke (Li et al., 2013; Mao et al., 2017). A recent research indicates that selective depletion of Tregs diminishes oligodendrogenesis and remyelination and impairs functional recovery after stroke (Shi et al., 2021). In addition, microglial depletion reverses the beneficial effects of transferred Tregs on white matter repair. Mechanistically, Tregs-derived osteopontin initiates a crosstalk between microglia and oligodendrocytes following stroke. Osteopontin triggers microglial polarization to the anti-inflammatory phenotype, thereby promoting oligodendrogenesis and white matter repair. IL-2/IL-2 antibody complex treatment also is able to improve white matter integrity and restore long-term functions by increasing endogenous Tregs numbers (Zhang et al., 2018). Collectively, Tregs might be a potential immune therapy for stroke rehabilitation. Double-negative T cells (DNTs; CD3^+^CD4^–^CD8^–^ T cell), distinguished from CD4^+^ T or CD8^+^ T cells, account for a small percentage (1 to 5%) of T cells in both mice and humans. The infiltrating DNTs are mainly located close to microglia in the ischemic brain of both stroke patients and mice. DNTs promote pro-inflammatory microglial activation after ischemic stroke and subsequently exacerbated brain injury (Meng et al., 2019; Wood, 2019).

### Non-pharmacological Interventions

#### Transcranial Focused Ultrasound Stimulation (tFUS)

Transcranial Focused Ultrasound Stimulation is a non-invasive neuromodulation technique, regulating the human somatosensory cortex (Lee et al., 2016). tFUS contributes to protection against ischemic brain damage (Li et al., 2017; Liu et al., 2019a; Wu et al., 2020). Recently, it is reported that ultrasound stimulation of the brain can modulate microglial polarization toward the anti-inflammatory phenotype, upregulate IL-10R and IL-10, and promote neurorehabilitation following stroke (Wang et al., 2021). However, whether the improvements in functional outcomes and neuroplasticity with ultrasound stimulation of the brain are mediated by microglial regulation after stroke remains unknown. Other rehabilitative approaches such as rTMS (Lin et al., 2021a), transcranial direct current stimulation (tDCS) (Zhang et al., 2020), and physical exercise (Nakanishi et al., 2021) have been demonstrated to induce anti-inflammatory microglial polarization. Whether their impacts on neuroplasticity after stroke are regulated by the inhibition of microglia-mediated neuroinflammation has not been understood. Hence, further studies are warranted to investigate these unanswered questions.

## Conclusion and Perspectives

For therapeutic strategies that target the primary ischemic brain injury, the neurodamage or neurodegenerative changes in the remote area should be taken into consideration. Neurogenesis and angiogenesis are coupled with the secondary neurodegenerative injury within the remote area, for example, the ipsilateral thalamus (Ling et al., 2009). Animal or other experimental study designs might need to assess the neurofunctional recovery, instead of just the primary injury area. A thorough understanding of the role of microglial regulation in stroke-induced plasticity globally is needed to achieve significant function recovery using combinatorial rehabilitation-related strategies targeting multiple pathways.

A thorough understanding of microglial activation in post-stroke brain plasticity and recovery is required: (1) Microglial activation exhibits different spatiotemporal manners in distinct animal models and human brains. A comprehensive understanding of such difference is warranted to investigate microglial targeted therapies. (2) Regulation of ion channel and surface receptor activity can instantaneously change microglial cell membrane voltage and function, which helps them rapidly adapt to acute stimuli (Izquierdo et al., 2019). However, how to switch microglia by manipulating specific microglial ion channels and/or surface receptors to favorable activation profile and coordinate them in the chronic stroke phase will definitely improve the neuroplastic recovery following stroke. (3) Systemic inflammation continues to activate the peripheral immune cells and inflammatory markers, which is associated with unfavorable outcomes in stroke patients. A thorough understanding of SIRS and potential treatment targets need to be investigated to improve the long-term functional outcomes. (4) New techniques distinguishing microglia and macrophages, especially when peripheral monocytes/macrophages infiltrate into the injured brain and exhibit various phenotypes, distinct activation roles, and cytokine production from such different cell populations, may drive different directions for the recovery. (5) Rodent animal and human brains have different microglial distribution profiles, which may exaggerate the translational gap. (6) Pathological conditions and baseline immunity in stroke patients might affect microglial activation, which also needs to be further investigated.

## Author Contributions

FY and TH searched the literatures and wrote the manuscript. YR, DL, and GT searched the literatures. LY provided the comments. JX designed this manuscript and provided the comments. ZL provided the idea and wrote the manuscript. All authors contributed to the manuscript and approved the submitted version.

## Conflict of Interest

The authors declare that the research was conducted in the absence of any commercial or financial relationships that could be construed as a potential conflict of interest. The reviewer SW declared a shared affiliation, with no collaboration, with the authors to the handling editor at the time of the review.

## Publisher’s Note

All claims expressed in this article are solely those of the authors and do not necessarily represent those of their affiliated organizations, or those of the publisher, the editors and the reviewers. Any product that may be evaluated in this article, or claim that may be made by its manufacturer, is not guaranteed or endorsed by the publisher.
